# Bif-1 deficiency impairs lipid homeostasis and causes obesity accompanied by insulin resistance

**DOI:** 10.1038/srep20453

**Published:** 2016-02-09

**Authors:** Ying Liu, Yoshinori Takahashi, Neelam Desai, Jun Zhang, Jacob M. Serfass, Yu-Guang Shi, Christopher J. Lynch, Hong-Gang Wang

**Affiliations:** 1 Department of Pharmacology, Penn State University College of Medicine, Hershey, PA 17033, USA; 2 Department of Pediatrics, Penn State University College of Medicine, Hershey, PA 17033, USA; 3 Department of Molecular Physiology, Penn State University College of Medicine, Hershey, PA 17033, USA; 4 Penn State Cancer Institute, Penn State University College of Medicine, Hershey, PA 17033, USA

## Abstract

Bif-1 is a membrane-curvature inducing protein that is implicated in the regulation of autophagy and tumorigenesis. Here, we report that Bif-1 plays a critical role in regulating lipid catabolism to control the size of lipid droplets and prevent the development of obesity and insulin resistance upon aging or dietary challenge. Our data show that Bif-1 deficiency promotes the expansion of adipose tissue mass without altering food intake or physical activities. While Bif-1 is dispensable for adipose tissue development, its deficiency reduces the basal rate of adipose tissue lipolysis and results in adipocyte hypertrophy upon aging. The importance of Bif-1 in lipid turnover is not limited to adipose tissue since fasting and refeeding-induced lipid droplet clearance is also attenuated by Bif-1 loss in the liver. Interestingly, obesity induced by a high fat-diet or Bif-1 deficiency downregulates the expression of proteins involved in the autophagy-lysosomal pathway, including Atg9a and Lamp1 in the adipose tissue. These findings thus identify Bif-1 as a novel regulator of lipid homeostasis to prevent the pathogenesis of obesity and its associated metabolic complications.

Obesity has become a worldwide health problem as a consequence of excessive high-calorie intake and the adoption of sedentary lifestyles. In 2012, nearly 17% of children and 35% of adults aged 20 years or older are obese in the US^1^. Obesity is associated with various health effects such as heart disease, type II diabetes, stroke, osteoarthritis, and several types of cancer^2^. However, controlling obesity and its associated health conditions remains a major challenge due to an incomplete understanding of its molecular machinery.

Recently, autophagy is emerging as a potential regulator of obesity. Autophagy is an evolutionarily conserved catabolic process, whereby cellular components are engulfed in a double-membrane structure, known as an autophagosome, and delivered to the lysosome for degradation^3^. Autophagy mediates the lipolysis of triacylglycerides (TAGs) stored in lipid droplets (LDs) in the liver, in a novel process referred to as lipophagy^4^. Bif-1 (SH3GLB1, Endophilin B1) is a member of the membrane curvature-driving endophilin protein family^5^ that positively regulates autophagosome formation by interacting with the class III phosphoinositide 3-kinase through ultraviolet irradiation resistant-associated gene (UVRAG) and regulating the trafficking of autophagy related protein 9 (Atg9)-containing vesicles^6^,^7^,^8^,^9^. Moreover, recent studies suggest that Bif-1 also regulates mitochondrial fission^10^, endocytic trafficking of epidermal growth factor receptor (EGFR)^11^ and tropomyosin receptor kinase A (TrkA)^12^, cytokinesis^13^, and coat protein complex I (COPI)-mediated retrograde trafficking from trans-Golgi network to endoplasmic reticulum^14^. Ablation of Bif-1 in mice does not cause any apparent developmental defects but increases the frequency of spontaneous tumorigenesis upon aging^6^. During our previous studies, we observed that Bif-1 knockout (KO) mice appeared more susceptible to body weight gain compared to their wild-type (WT) littermates. In line with this observation, Bif-1 is highly expressed in human adipose tissue^15^,^16^, suggesting the potential importance of Bif-1 in the regulation of fat storage.

In the present study, we provide evidence that Bif-1 serves a novel function in controlling the degradation of lipid droplets. Bif-1-deficient mice progress to obesity and develop hyperinsulinemia upon aging or a long-term high fat-diet (HFD) challenge.

## Results

### Ablation of Bif-1 promotes aging- and diet-induced weight gain and fat accumulation

**Figure 1:**
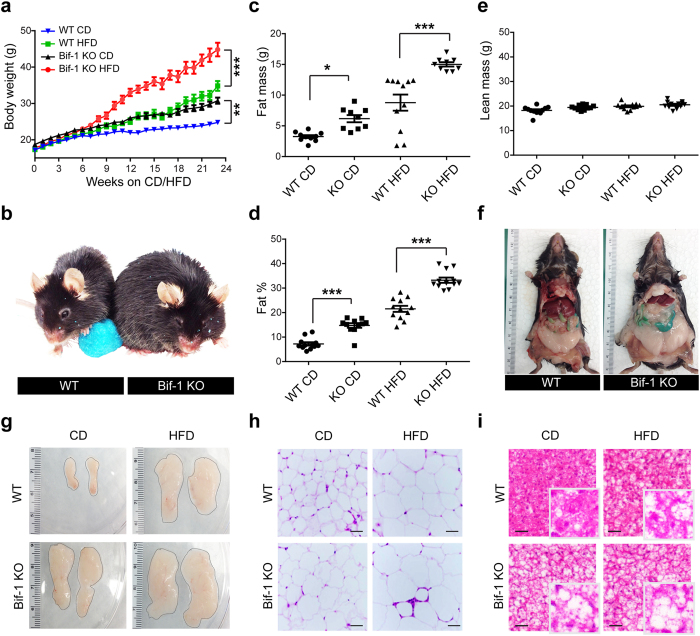
Loss of Bif-1 induces obesity. (**a**) Female wild type (WT) and Bif-1 knockout (KO) mice were fed with either control chow diet (CD) or high-fat diet (HFD) for the indicated periods of time. Body weight of each mouse was monitored weekly (*n* = 9–12). Statistical significance was determined using one-way ANOVA followed by Bonferroni’s multiple comparison test. (**b**) Representative images of WT and Bif-1 KO mice fed on HFD for 23 weeks. (**c–e**) Body composition of mice fed with the indicated diet for 23 weeks was measured using TD-NMR. Loss of Bif-1 leads to larger total (**c**) and percent (**d**) fat mass and has no effect on lean mass (**e**). Statistical significance was determined using one-way ANOVA followed by Bonferroni’s multiple comparison. (**f**) Representative images of fat deposits in WT and Bif-1 KO mice fed on HFD for 23 weeks. (**g**) Representative images of abdominal gonadal fat pads from WT and Bif-1 KO mice fed on either CD or HFD for 23 weeks. (**h**,**i**) Loss of Bif-1 leads to adipocyte enlargement. Hematoxylin and eosin stain of the abdominal gonadal white adipose tissue (WAT) (**h**) and interscapular brown adipose tissue (**i**) sections from mice fed with CD or HFD for 23 weeks. Scale bars in (**h**) and (**i**) represent 50μm and 200μm, respectively. All values are mean ± SEM. Differences with controls were significant for *p < 0.05, **p < 0.01, and ***p < 0.001.

To assess whether Bif-1 is critical in regulating obesity, we placed WT and Bif-1 KO mice of the C57BL/6 background on a high fat diet (HFD) or a control/standard chow diet (CD) starting at six weeks of age, at which time the mice were indistinguishable by weight. Consistent with our previous observations, Bif-1 KO mice gained more weight upon aging than their WT littermates (Fig. 1a; Supplementary Fig. S1), which was notably exacerbated with the HFD challenge (Female WT: 34.83 ± 1.36 g, Bif-1 KO: 44.83 ± 1.87 g, Fig.1a,b; Male WT: 40.44 ± 2.03 g, Bif-1 KO: 51.14 ± 0.76 g, Fig. 1a; Supplementary Fig. S1). Significant weight gain occurred as early as one week after HFD feeding in males and eight weeks in females, which is in line with the previous observation that female mice are less sensitive to HFD-induced obesity^17^.

To determine whether the higher body weight gain in Bif-1 KO mice correlates with increased fat accumulation, we analyzed the full body composition of these mice at study end-points (19 and 23 weeks after the initiation of dietary challenge for males and females, respectively). Bif-1 KO mice showed a significant increase in the absolute and relative amount of body fat compared to WT mice regardless of diet type (Fig. 1c,d; Supplementary Fig. S1). In contrast, lean mass (Fig. 1e; Supplementary Fig. S1) and body water content (data not shown) were comparable between the WT and Bif-1 KO mice fed with CD. While a moderate increase in lean mass was observed in male Bif-1 KO mice after prolonged HFD challenge (Supplementary Fig. S1), the increase was not nearly as significant as that observed in fat mass. In agreement with these observations, we observed significantly larger abdominal gonadal and subcutaneous fat pads (Fig. 1f,g) as well as enlargement of white adipocytes (Fig. 1h) in Bif-1 KO mice. Similarly, the brown adipose tissue of Bif-1 KO mice also appeared to contain more fat than their WT littermates (Fig. 1i). We did not observe apparent ectopic fat accumulation in peripheral tissues such as liver and muscle within the time span of the current study. Taken together, these results indicate that Bif-1 deficiency increases adiposity upon aging and promotes obesity after a HFD challenge.

### Development of insulin resistance in Bif-1 KO mice following excessive adiposity

**Figure 2:**
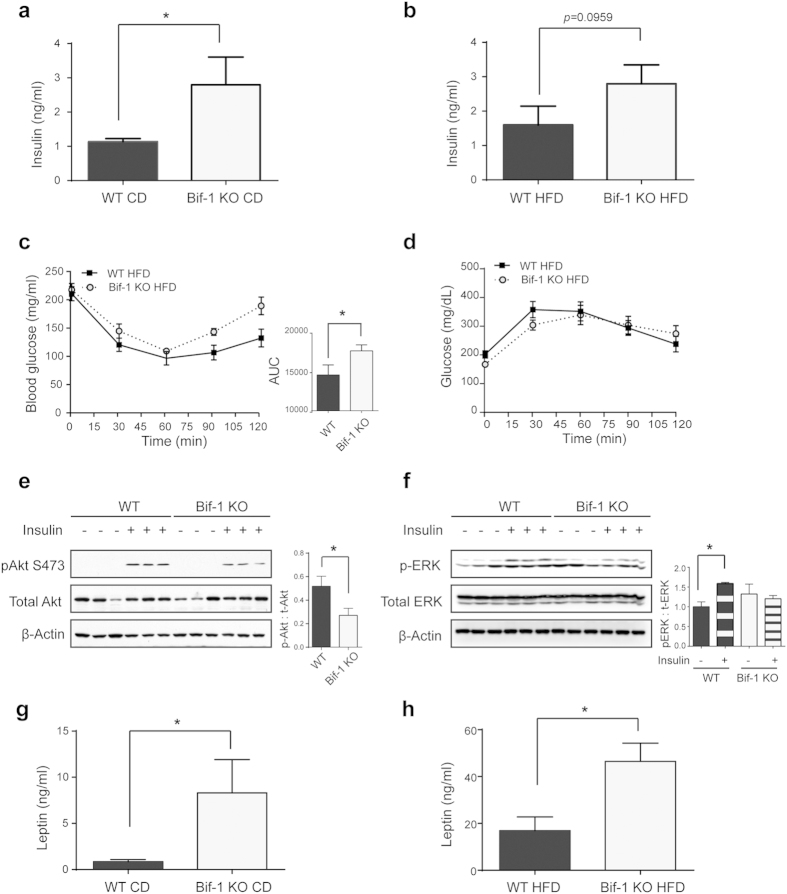
Obesity induced by Bif-1 loss leads to insulin resistance. (**a**,**b**) Levels of insulin in plasma from mice fed with CD (**a**) or HFD (**b**) for 23 weeks were measured using BioRad Luminex assays, n = 9–12. Statistical significance was determined using Student’s *t*-test. (**c**,**d**) Insulin tolerance test (**c**) and oral glucose tolerance test in mice fed with HFD for 23 weeks were performed as described in Methods. *n* = 9–11. Statistical significance was determined using Student’s *t*-test. (**e**,**f**) WT and Bif-1 KO mice fed with CD for 23 weeks were fasted for 16 h and then given 0.75 U/kg of insulin intraperitoneally 15 min prior to sacrifice. Liver (**e**) and gastric muscle (**f**) lysates were prepared and subjected to immunoblot analysis using the indicated antibodies. The intensities of phosphorylated-Akt (pAkt S473) or phosphorylated-ERK_1/2_ (p-ERK) relative to total Akt or total ERK_1/2_ were normalized against β-actin. Statistical significance was determined using Student’s *t*-test in (**e**) and One-way ANOVA followed by Bonferroni’s multiple comparison test in (**f**). (**g**,**h**) Levels of leptin in plasma from mice fed with CD (**g**) or HFD (**h**) for 23 weeks were measured using BioRad Luminex assays, n = 9–12. Statistical significance was determined using Student’s *t*-test. All values are mean ± SEM. Differences with controls were significant for *p < 0.05.

Obesity causes insulin resistance^18^,^19^,^20^; therefore higher adiposity in Bif-1 KO mice suggests that these animals may be less responsive to insulin. To investigate the possibility of such metabolic complications, we first measured the circulating insulin levels in the mice. We found that Bif-1 KO mice fed with CD had normal insulin levels at 10 weeks of age at which time their body weight had not yet diverged from WT littermates (Supplementary Fig. S2). However, upon weight augmentation by aging or diet challenge (30 weeks old), the Bif-1 KO mice showed hyperinsulinemia (Fig. 2a,b) and similar circulating glucose levels compared to the WT mice (data not shown), indicating that the obese Bif-1 KO mice had lower insulin sensitivity. Consistently, Bif-1 KO mice displayed intensified insulin resistance after HFD challenge compared to the WT mice (Fig. 2c), although glucose tolerance was not significantly affected (Fig. 2d). Moreover, Bif-1 KO mice (30 weeks old, CD fed) displayed dampened insulin signaling in the liver compared to WT mice, as determined by a moderate decrease in phosphorylated-Akt (p-Akt) in response to insulin injection (Fig. 2e). We were unable to detect p-Akt in gastric muscle lysates collected from the insulin-treated mice. We thus used the phosphorylation of ERK (p-ERK) as an alternative means for detecting insulin signaling. While the WT muscle displayed elevated levels of p-ERK following insulin injection, the Bif-1 KO muscle did not show such induction of signaling (Fig. 2f). Notably, we also observed hyperleptinemia in Bif-1 KO mice upon the development of obesity (Fig. 2g,h, Supplementary Fig. S2), which further demonstrates that Bif-1 is important for the suppression of obesity-associated metabolic complications.

### Bif-1 does not regulate food intake, physical activities or fuel preference

**Figure 3:**
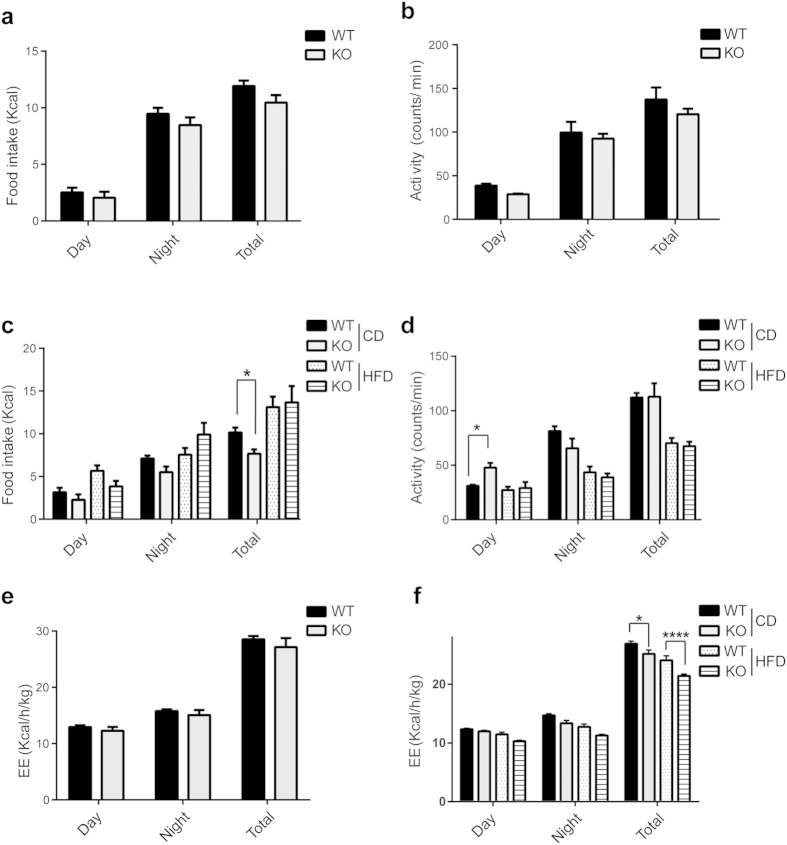
Effects of Bif-1 on food intake, physical activities, and fuel preference. Indirect calorimetry analysis of WT and Bif-1 KO mice fed with CD for 4 weeks (10-week old, n = 4) (**a**,**b**,**e**) or with the indicated diet for 19 weeks (25-week old, n = 8) (**c**,**d**,**f**). Food intake (**a**,**c**), physical activities (**b**,**d**), and energy expenditure (**e**,**f**) were measured during a 48-h cycle using metabolic cages. Statistical significance was determined using one-way ANOVA followed by Bonferroni’s multiple comparison. All values are mean ± SEM. Differences with controls were significant for *p < 0.05 and ****p < 0.0001.

Weight gain is a result of imbalance in energy intake versus expenditure; thus we analyzed the food intake and energy metabolism of the WT and Bif-1 KO mice using metabolic cages. In order to eliminate the confounding effects due to obesity (e.g. hyperleptinemia), we analyzed WT and Bif-1 KO mice both before and after their body weights diverged. Food intake and physical activities were comparable between WT and Bif-1 KO mice at a young age (10 weeks old, CD fed) (Fig. 3a,b), suggesting that these two factors are not the cause of increased obesity in Bif-1 KO mice. Upon weight augmentation by aging (27 weeks old), the total amount of food intake in CD-fed Bif-1 KO mice was lower than that in the WT littermates (Fig. 3c). This is in agreement with a previous finding that augmentation of fat mass leads to a graded decrease in voluntary feeding as a feedback mechanism of the body to favor a reduction in fat stores^21^. Moreover, the 27-week-old Bif-1 KO mice fed with CD were slightly more physically active during the day relative to the nighttime compared to the WT, suggesting a mild impairment of circadian rhythms (Fig. 3d).

As Bif-1 is involved in the maintenance of mitochondrial integrity, we next asked whether Bif-1 deficiency compromises the mitochondrial fatty acid oxidation. To answer this question, we analyzed the respiratory exchange ratio (RER) in 10-week-old WT and Bif-1 KO mice. RER is the ratio between the amount of oxygen (O_2_) consumed and carbon dioxide (CO_2_) produced, and is an indicator of which energetic substrate is being oxidized to supply the body with energy^22^. RER is 1.0 for carbohydrates and 0.7 for lipids. As shown in Supplementary Figure S3, both the WT and Bif-1 KO mice had RER values slightly above 0.8 during the day, which equally increased to close to 1 during the night. These results indicate that Bif-1 deficiency does not affect the animals’ fatty acid oxidation, and WT and Bif-1 KO mice can oxidize a mix of carbohydrates and fat with similar efficiency. Consequently, the WT and Bif-1 KO mice had comparable energy expenditure at 10 weeks of age (Fig. 3e). Finally, aging or long-term HFD feeding significantly reduced the energy expenditure of Bif-1 KO mice compared to WT controls (Fig. 3f), thereby indicating a positive energy balance and excessive energy store in the animals at the obese stage.

### Development of obesity with Bif-1 deficiency is independent of adipogenesis

**Figure 4:**
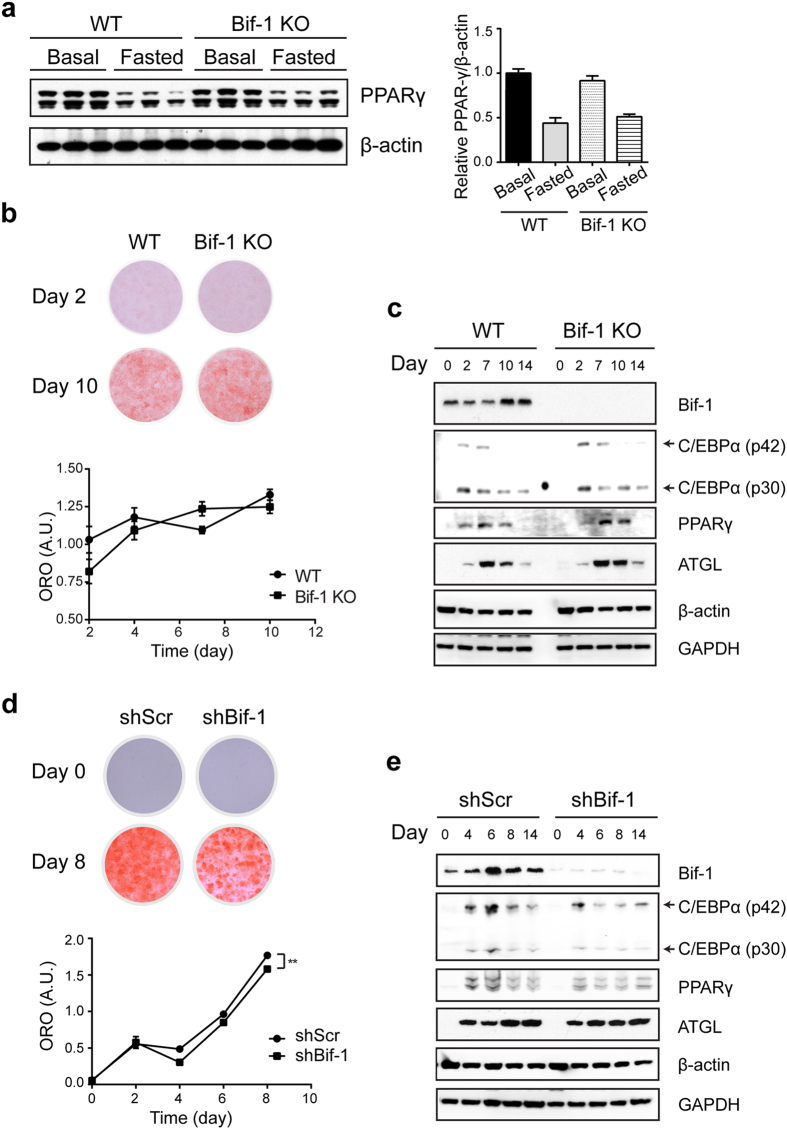
Bif-1 deficiency does not affect adipogenesis. (**a**) Bif-1 does not impact adipogenesis *in vivo*. Relative protein expression levels of PPAR-γ in WT and Bif-1 KO WAT homogenates prepared from 6- week-old mice were determined *via* immunoblotting (n = 3). (**b**) Immortalized perivascular cells isolated from brown adipose tissue (iPBA) of 6-week-old mice as described in Methods were induced to differentiate with an adipogenic cocktail. Cells were subjected to Oil Red O (ORO) staining on the indicated days following adipogenesis induction. Representative images were acquired, and the optical density of ORO extracted from the cells was quantified by absorption at 520 nm (n = 4). (**c**) Lysates prepared from iPBA cells on the indicated days during adipogenesis were subjected to immunoblotting with the indicated antibodies. (**d**) 3T3-L1 cells transduced with lentiviruses encoding Bif-1 shRNA (shBif-1) or scrambled shRNA (shScr) were induced to differentiate as described in Methods Cells on indicated days during adipogenesis were stained with ORO. Representative images were acquired, and the optical density of ORO extracted from the cells was quantified by absorption at 520nm (n = 4). Statistical significance was determined using Student’s *t*-test. All values are mean ± SEM. Differences with controls were significant for **p < 0.01. (**e**) Lysates prepared from 3T3-L1 shScr and shBif-1 cells on the indicated days during adipogenesis were subjected to immunoblotting with the indicated antibodies.

Obesity is characterized by an expansion of adipose tissue mass. To determine the potential importance of Bif-1 in adipogenesis, we first examined the protein expression of peroxisome-proliferator activated receptor gamma (PPARγ), a master regulator of adipogenesis, in the white adipose tissue (WAT) of 6-week-old WT and Bif-1 KO mice. However, we did not observe any apparent difference between the two genotypes under both basal and nutrient-deprived states (Fig. 4a). Consistently, an *in-vitro* adipogenesis assay using immortalized peri-vascular cells isolated from the brown adipose tissue of the WT and Bif-1 KO mice did not reveal a significant difference in adipogenesis, as indicated by comparable intensities of Oil Red O stain and protein expression levels of adipogenesis markers after stimulation with an adipogenic cocktail (Fig. 4b,c). This similarity in the adipogenic potential between the WT and Bif-1 KO peri-vascular cells was also confirmed using 3T3-L1 cells (Fig. 4d,e). Interestingly, Bif-1 expression was dramatically increased upon the induction of PPARγ expression, which is in line with a recent report showing that Bif-1 is one of the target genes of PPARγ^23^. Taken together, our results suggest that obesity induced by the loss of Bif-1 is not likely due to the promotion of adipogenesis.

### Bif-1 loss impairs lipid turnover in adipose tissue and liver

**Figure 5:**
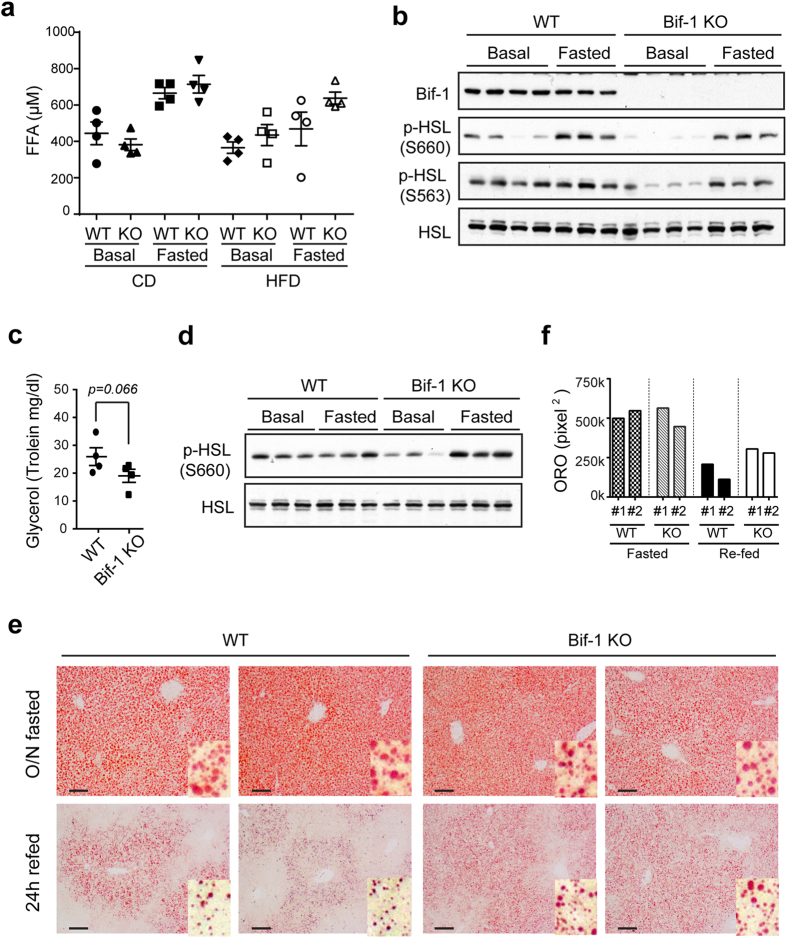
Bif-1 regulates lipid turnover in adipose tissue and liver. (**a**) Plasma levels of free fatty acid (FFA) from mice fed with the indicated diet for 23 weeks in resting and 16 h-fasted states were measured (*n* = 4). (**b**) Lysates prepared from white adipose tissue collected from the same animals in (**a**) were subjected to immunoblotting with the indicated antibodies. Same lysates were also used in Fig. 6b. (**c**) Plasma levels of glycerol from 6-week old mice fed with normal CD were measured using glycerol reagent (Sigma). Statistical significance was determined using Student’s *t*-test. (**d**) Lysates prepared from white adipose tissue collected from the same animals in (**c**) were subjected to immunoblotting with the indicated antibodies. Same lysates were also used in Fig. 4a. (**e**,**f**) Representative images (**e**) and quantification (**f**) of ORO stain of the liver sections from mice fed with CD for 20 weeks, fasted overnight or fasted overnight and then refed for 24 h. Scale bars represent 100 μm.

As Bif-1 deficiency does not enhance adipogenesis, we next determined whether Bif-1 is important for lipolysis or the mobilization of lipids from WAT. At the resting state, WAT triacylglycerol (TAG) is in a constant state of flux as a result of a largely futile cycle of lipolysis and fatty acid re-esterification^24^. During times of energy deprivation, the WAT enhances the rate of lipolysis to hydrolyze TAG to free fatty acids (FFAs) and glycerol that are released into the circulation and taken up by other organs as energy substrates. The best known mechanism of fasting-induced lipolysis involves the activation of PKA upon the binding of catecholamines to the β_2_-adrenergic receptors, which in turn phosphorylates and activates hormone sensitive lipase (HSL). To examine whether Bif-1 regulates lipolysis, we measured the FFA levels in the plasma of the 30-week-old mice under basal and fasting states. Interestingly, loss of Bif-1 appeared to cause a slight decrease in plasma FFA levels during resting state but not after fasting or a long term HFD challenge (Fig. 5a). This decrease in FFA is well correlated with a reduction in the protein expression levels of phosphorylated-HSL (p-HSL) in the Bif-1 KO mice under basal state (Fig. 5b). This suggests that Bif-1 may contribute to basal lipolysis but is likely not important for fasting-induced lipolysis. Therefore, Bif-1 KO mice could provide a sufficient amount of FFAs and glycerol to meet their energy demand during short-term fasting. Importantly, we observed a moderate decrease in the plasma glycerol levels and protein levels of p-HSL in young animals when their body weights had not diverged (Fig. 5c,d, Supplementary Fig. S4), suggesting that this impairment in basal lipolysis is not caused by development of obesity.

In addition to the examination of lipolysis in WAT, we also determined whether Bif-1 is important for lipid breakdown in the liver, which is the major site of lipid synthesis and temporary lipid storage. We subjected the mice to overnight fasting and then re-fed the animals for another day. During fasting, FFAs generated by adipose tissue lipolysis are transported to the liver where they are re-esterified to form triglycerides, which are then degraded upon re-feeding. While Bif-1 deficiency did not affect the initial accumulation of hepatic lipid droplets upon fasting, lipid droplets were significantly increased in number and size in Bif-1 KO mice following refeeding (Fig. 5e,f), suggesting that Bif-1 is important for hepatic lipolysis in the presence of excessive lipids. Taken together, these results suggest that Bif-1 regulates lipid catabolism not only in the adipose tissue, but also in other peripheral tissues.

### Bif-1 deficiency reduces autophagic flux and downregulates autophagic-lysosomal proteins

**Figure 6:**
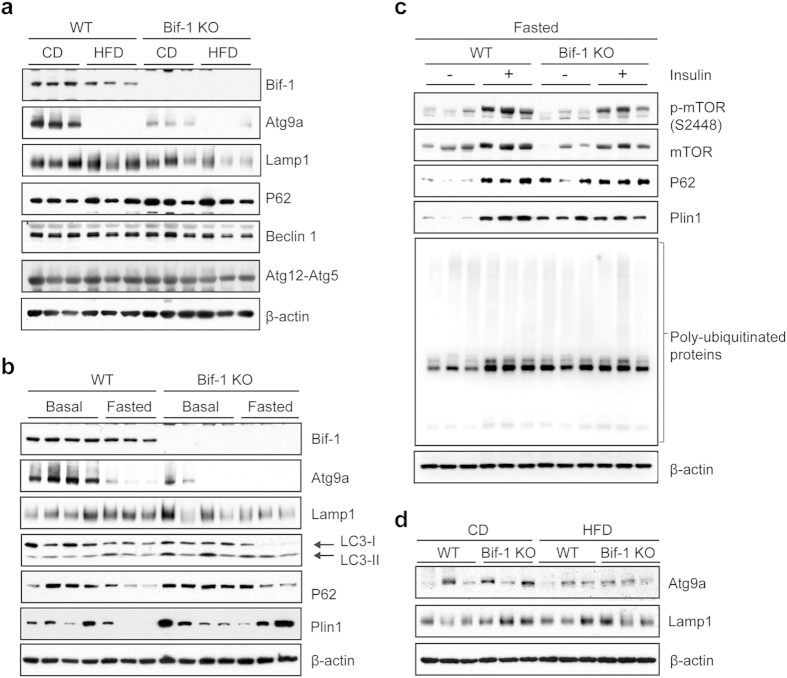
Bif-1 is important for autophagy in adipose tissue. (**a**–**c**) Immunoblots for the indicated proteins in WAT homogenates prepared from 30-week-old mice. The mice were fed with CD or HFD for 23 weeks (**a**), fed with CD, at basal or 16 h-fasted states (**b**), or fasted for 16 h, with or without insulin injection 15 min prior to sacrifice (**c**). n = 3–4. (**d**) Immunoblots for the indicated proteins in WAT homogenates from 6-week old mice fed with CD or 11-week old mice fed with 5 weeks HFD. n = 3.

It has been recently discovered that autophagy can directly degrade lipid droplets in hepatocytes. Since then, a plethora of other studies have also identified lipophagy as a conserved mechanism to mobilize cellular fat stores in diverse cell types^25^,^26^,^27^,^28^. However, the role of autophagy in adipose tissue remains unclear. We thus evaluated the expression levels of p62 and LC3-II, which are widely used markers for autophagy^29^. P62 serves an autophagic cargo receptor and is itself a substrate for autophagy^29^. LC3-II marks autophagosomal membrane turnover and is generated through the conjugation of its precursor, LC3-I, to the membrane lipid phosphatidylethanolamine via a series of ubiquitin-like conjugation reactions^3^. In our study we observed that compared to WT, Bif-1 deficient adipose tissue had slightly higher levels of p62 when fed with CD under both basal and fasting states (Fig. 6a,b, Supplementary Fig. S5), indicating a decrease in autophagic flux. A two-fold increase in the LC3-II/LC3 ratio was detected in WT adipose tissue upon fasting, while the Bif-1 deficient adipose tissue showed a minor elevation in LC3-II/LC3 ratio, indicating a dampened autophagic response. Interestingly, we found that in WT animals a long-term HFD challenge significantly suppressed the expression of Bif-1 and Atg9a, which is another essential autophagy protein (Fig. 6a, Supplementary Fig. S5). This Atg9a reduction was phenocopied by the CD-fed Bif-1 KO mice (Fig. 6a,b, Supplementary Fig. S5). Similarly, the expression of lysosomal-associated membrane protein 1 (Lamp1) was also reduced in Bif-1 KO adipose tissue compared to WT regardless of diet type or nutrition status (Fig. 6a,b, Supplementary Fig. S5). As the downregulation of Atg9 and Lamp1 in Bif-1 KO adipose tissue was only observed in 30-week-old but not 6-week- or 10-week-old animals (Fig. 6d, Supplementary Fig. S5), this effect appears to be a consequence of the development of obesity in Bif-1 KO mice. The expression levels of other Atg proteins, including Beclin 1 and the Atg12-Atg5 conjugate, did not appear to be affected by Bif-1 expression (Fig. 6a). Interestingly, expression of perilipin A (Plin1), a lipid droplet coating protein, is also slightly higher under both basal and fasting states in the WAT of Bif-1 KO mice, which correlates with the reduced TAG hydrolysis observed in these animals (Fig. 5a). To further assess the potential role of Bif-1 and autophagy in the regulation of Plin1 in adipose tissue, a portion of fasted WT and Bif-1 KO mice were injected with insulin to activate mTOR signaling and inhibit autophagy^30^. Insulin administration led to the accumulation of p62 in WT adipose tissue, indicating rapid autophagy flux in WAT following fasting, and potent suppression of autophagy by insulin (Fig. 6c, Supplementary Fig. S5). Notably, Plin1 also accumulated upon insulin treatment in WT adipose tissue, indicating a role for autophagy in Plin1 degradation. In contrast, the level of p62 and Plin1 in fasted Bif-1 KO mice was not further increased by insulin (Fig. 6c, Supplementary Fig. S5), suggesting an impairment of autophagy under fasting conditions in these mice.

## Discussion

Autophagy is a critical housekeeping pathway that controls cellular and organismal metabolism by degrading proteins, lipids and organelles^31^. This catabolic process has been shown to be suppressed in adipocytes in human and rodent obesity^32^. However, the role of autophagy in adipose tissue fat catabolism remains unclear. In this study, we show that Bif-1 deficiency induces adipocyte hypertrophy without altering food intake and physical activity and promotes the development of obesity and insulin resistance upon aging or dietary challenge in mice. The observations that Bif-1 loss decreases the levels of phosphorylated HSL and plasma concentration of glycerol under fed condition suggest an important role of autophagy in the promotion of basal lipolysis in the WAT. Interestingly, unlike in the fed condition, Bif-1 deficiency does not block adipose tissue lipolysis and LD formation in the liver during fasting, suggesting that autophagy may be dispensable for hormone-stimulated lipolysis. This observation is consistent with a recent report showing that lysosomal inhibition has a minimal effect on the β-adrenergic agonist isoproterenol-stimulated lipolysis *in vitro*^32^. Therefore, Bif-1 regulates basal adipose tissue lipolysis through a mechanism that is independent of the catecholamine-induced canonical lipolytic pathway. Since the hepatic lipid droplet clearance induced by fasting followed by refeeding was also found to be suppressed by Bif-1 deficiency, the importance of Bif-1-mediated lipid turnover may not be limited to adipose tissue.

Under basal conditions, lipid droplet-coating Plin1 limits the access of HSL to prevent triacylglycerol hydrolysis^33^, while its degradation facilitates lipolysis^34^. Our data show that Bif-1 deficiency impairs turnover of Plin1, autophagic substrate p62, and poly-ubiquitinated proteins in WAT. This observation is in agreement with a previous finding that autophagy inhibition compromises the ubiquitin-proteasome system^35^. Since the degradation of Plin1 is mediated through the ubiquitin-proteasome^36^ and the lysosomal^34^ pathways, inhibition of overall Plin1 degradation could be the mechanism by which Bif-1 loss attenuates basal lipolytic activities in adipose tissue. Alternatively, or in addition, Bif-1 may regulate adipose tissue lipid catabolism by promoting lipophagy; whereby portions or entire LDs are engulfed into autophagosomes and delivered to lysosomes for degradation^4^. Indeed, our data show that the induction of autophagy by fasting promotes the degradation of Plin1 in a Bif-1-dependent manner. However, it is unlikely that lipophagy plays an essential role in adipose tissue lipid catabolism since, as described above, Bif-1 deficiency does not inhibit adipose tissue lipolysis upon fasting. Moreover, in contrast to basal conditions^33^, Plin1 has been shown to facilitate lipolysis by recruiting HSL on the surface of LDs under the exposure to β-adrenergic agonist isoproterenol^37^, which mimics fasting conditions. Recently, chaperone-mediated autophagy (CMA) has been shown to selectively degrade Plin2 and Plin3 to control the access of adipose tissue triglyceride lipase and several key autophagy-related proteins in fibroblasts^38^. It would be interesting to determine if the Bif-1-mediated autophagic pathway collaborates with CMA to regulate basal lipolysis in adipose tissue.

Our data also show that the expression levels of Atg9a and Lamp1 in WAT are reduced by the loss of Bif-1 upon aging or dietary challenge. Downregulation of these two critical proteins for autophagy could in turn undermine the autophagic activity on LD and promote further fat accumulation. Although further studies are required to understand the mechanism responsible for the downregulation of these autophagy-lysosomal proteins, recent studies have shown that Lamp1 and Atg9 genes are targets of the transcription factor EB (TFEB)^39^ which is negatively regulated by mTOR^40^. Therefore obesity-attributed hyperinsulinemia in Bif-1 KO mice may mediate the downregulation of Atg9 and Lamp1 through the activation of mTOR and suppression of TFEB signaling, and this hypothesis warrants further investigation in the future.

In summary, the current work demonstrates the importance of Bif-1 in the control of lipid catabolism and whole body energy and nutrient homeostasis to prevent the development of obesity. As excessive lipolysis in turn increases the risk of developing obesity-associated comorbidities, controlling the level of Bif-1 mediated lipolysis could be an important factor for obesity therapy. It is worthwhile to mention that unlike Atg5 and Atg7^41^,^42^, *Bif-1* loss does not affect adipogenesis *in vivo*. The precise mechanism of how Atg5 and Atg7 promote adipogenesis is unclear, but it could be mediated through the LC3 conjugation system, which is critical for the LD biogenesis in a manner that seems to be independent of its function in autophagy^43^. In this regard, Bif-1 is not absolutely required for the LC3 conjugation process^6^,^7^,^8^. As the expression of Bif-1 is upregulated upon adipogenesis but declined in obesity, Bif-1 deficient mice may be a suitable model to study the roles of adipose tissue autophagy in obesity-associated metabolic complications.

## Methods

### Reagents

The following antibodies were used in this study: polyclonal goat anti-Bif-1 (GTX 21343, GeneTex); poly-clonal guinea pig anti-P62 (03-GP62-C, American Research Products); monoclonal rabbit anti-Plin1 (9349, Cell Signaling); polyclonal rabbit anti-Atg9a (GTX128427, Genetex); monoclonal rat anti-Lamp1 (19992, Santa Cruz); polyclonal rabbit anti-phospho-mTOR (S2448) (2971, Cell Signaling); monoclonal rabbit anti-mTOR (2983, Cell Signaling); monoclonal rabbit anti-phospho-Akt (S473) (9271, Cell Signaling); monoclonal mouse anti-Akt (MAB2055, R&D Systems); monoclonal rabbit anti-PPARγ (2443, Cell Signaling); monoclonal rabbit anti-phospho-p44/42 MAPK (Erk1/2) (Thr202/Tyr204) (4370, Cell Signaling); monoclonal rabbit anti-p44/42 MAPK (Erk1/2) (9102, Cell Signaling); polyclonal rabbit anti-LC3B (NB100-2220, Novus Biologicals); monoclonal mouse anti-Atg5 (M153-3, MBL); polyclonal goat anti-Beclin1 (sc-10086, Santa Cruz); monoclonal mouse anti-Ubiquitin (NP300-130, Novus Biologicals); monoclonal mouse anti-β actin (A5441, Sigma). Mouse leptin ELISA kit and mouse insulin ELISA kit were purchased from Millipore. Mouse diabetes Luminex multiplex assay kit was purchased from Bio-Rad. Fatty acid kit was purchased from Cayman Chemicals. Unless otherwise stated, all other chemicals were purchased from Sigma.

### Mice

Generation of wild type Bif-1+/+ mice and whole body Bif-1−/− mice in C57BL/6 background was described previously^8^. For dietary challenge experiments, 6-week-old mice were fed with standard chow providing 18% calories from fat (Harlan Teklad, TD2918) or a HFD providing 55% calories from fat (Harlan Teklad, TD93075). Mouse studies were conducted in accordance with federal guidelines and were approved by the Pennsylvania State University Animal Care and Use Committee.

### Body composition, energy expenditure, activity, and food intake

Fat and lean body mass of each mouse were analyzed on an LF90 TD-NMR (Bruker Optics). For measuring metabolic parameters, mice were acclimated in the metabolic chambers (TSE Systems) for 2 days, and food and water intake, energy expenditure, and physical activities were measured for another 2 consecutive days. Constant airflow (0.2 l/min) was drawn through the chamber and the concentrations of oxygen and carbon dioxide were monitored at the inlet and outlet of the sealed chambers to calculate oxygen consumption. Each chamber was measured for 3.75 min at 30 min intervals. Physical activity was monitored by infrared technology (OPT-M3, Columbus Instruments), as the count of 3D beam breaking (X total, Y ambulatory, and Z) was measured.

### Oral glucose tolerance test (OGTT) and insulin tolerance test (ITT)

OGTT and ITT were performed in overnight and 9 h-fasted mice, respectively. Glucose was delivered by oral gavage at 2.5 g/kg body weight after initial measurement of fasting blood glucose. Insulin was delivered by intraperitoneal injection (0.75 U/kg body weight; Novolin, Novo Nordisk). Blood glucose was determined at 0, 30, 60, and 120 min after the glucose or insulin administration with an Embrace blood glucose meter (Omnis Health).

### *In-vitro* adipogenesis in immortalized perivascular cells

Perivascular cells were isolated from brown adipose tissue of 10-week-old WT and Bif-1 KO mice fed with CD. Briefly, interscapular brown fat pad was isolated from mice, dissected and minced in PBS. Minced tissue was then pipetted into isolation buffer containing 123.0 mM NaCl, 5.0 mM KCl, 1.3 mM CaCl_2_, 5.0 mM Glucose, 100 mM HEPES, 1% pen/strep , 4% BSA, and 1.5 mg/ml type I collagenase. The mixture was then vortexed for 10 seconds, and incubated at 37 degrees for 40 min with gentle shaking. Digested tissue was then filtered using 100 μm Nylon cell strainers into clean Eppendorf tubes, and centrifuged at 1500 rpm for 5 min. Pellet was re-suspended in culture medium (DMEM medium supplemented with 20% fetal bovine serum (FBS), 20 mM HEPES, and 1% pen/strep), and cultured overnight to allow cells to attach. Primary cells were then immortalized with SV40 large T antigen and used for adipogenesis experiments. To induce cell differentiation, immortalized peri-vascular cells were seeded at 5000 cells/cm^2^ in culture dishes and grown to confluence in culture medium. Confluent cells were then switched to differentiation media containing 20% FBS, 20 nM insulin, and 1 nM triiodothyronine for another 48 hours. Adipogenesis was induced by treating cells for 48 h in differentiation medium that was further supplemented with 0.5 μM dexamethasone, 0.5 mM isobutylmethylxanthine (IBMX), and 0.125 mM indomethacin. After induction, cells were returned to differentiation medium, which was replenished every 2 days.

### *In-vitro* adipogenesis in 3T3-L1 cells

3T3-L1 cells were transduced with lentiviruses encoding Bif-1 shRNA (shBif-1) or control scrambled shRNA (shScr) and induced to differentiate with a standard adipogenic cocktail. Briefly, cells were seeded at 5000 cells/cm^2^ in culture dishes and grown to confluence in culture medium (DMEM medium supplemented with 10% bovine calf serum and 1% pen/strep). Cells were then cultured in fresh culture medium for another 2 days. At two days post-confluence (designated day 0), cells were induced to differentiate in induction medium (DMEM supplemented with 10% FBS, 1% pen/strep, 1 μM dexamethasone, 0.5 mM IBMX, and 10 μg/ml insulin). After 3 days induction, the media were replaced with maintenance medium (induction medium minus IBMX) which was replenished every 2 days during the rest of differentiation time course.

### Oil Red O (ORO) stain and quantification

ORO staining solutions were prepared following standard protocols. Briefly, for staining cells, a 3.5 mg/ml stock solution was made by dissolving ORO powder in isopropanol. Immediately before stain, fresh working solution was prepared by mixing 6 parts of stock solution with 4 parts of distilled H_2_O. The mixture was incubated at room temperature for 20 min and then filtered through 0.2 μm filters. Cells were fixed with 4% paraformaldehyde for 10 min, and washed with 60% isopropanol for 5 min × 2 times. Cells were briefly air-dried, and stained with ORO working solution for 30 min at room temperature. Cells were washed with PBS for 5 min × 3 times and pictures of stained dishes were acquired using a digital camera. To quantify stain, ORO in cells was extracted with 4% NP-40 in isopropanol and subjected to optical density measurement at 520nm. For staining liver tissue, a 2.5 mg/ml ORO stock solution was made in isopropanol. Immediately before tissue staining, working solutions were prepared by mixing 6 parts of stock solution and 4 parts of distilled H_2_O. The mixture was incubated at 4 degree for 5 min, and filtered through 0.45 μm filters. 12-μm-thick liver sections from frozen tissue were fixed with 4% paraformaldehyde, and stained with the ORO working solution for 30 min at room temperature. Bright field microscopic images were acquired, and quantification of ORO stain was performed using Image J software with a Threshold Colour plugin. Briefly, images were converted to 8-bit type with default colour thresholding. Total area (pixel^2^) of ORO-positive particles was then analyzed for the entire image. Images for at least two mice per condition were used for quantification.

### Fatty acid measurement

Blood was collected from mice through cardiac puncture immediately after enthanization, and centrifuged at 1300 xg at room temperature to obtain plasma. Plasma levels of fatty acid were then measured using fatty acid kit purchased from Cayman Chemicals.

### Statistical test

All data are expressed as mean ± S.E.M. Student *t*-tests were used for single comparisons. One-way ANOVA tests were performed followed by Bonferroni’s or Dunn’s post-hoc tests for multiple comparisons unless specified. Statistical significance is assumed at *p < 0.05, **p < 0.01, ***p < 0.001, and ****p < 0.0001. Sample size was chosen based on results from pilot studies.

## Additional Information

**How to cite this article**: Liu, Y. *et al*. Bif-1 deficiency impairs lipid homeostasis and causes obesity accompanied by insulin resistance. *Sci. Rep*. **6**, 20453; doi: 10.1038/srep20453 (2016).

## Supplementary Material

Supplementary Information
