# Supplementary material for: Bif-1 deficiency impairs lipid homeostasis and causes obesity accompanied by insulin resistance
Source: Sci Rep. 2016 Feb 9;6:20453. doi: 10.1038/srep20453 (PMC4746598; doi:10.1038/srep20453)
Supplement: Supplementary Information [file srep20453-s1.pdf]

Bif-1 deficiency impairs lipid homeostasis and causes obesity accompanied by insulin resistance

Ying Liu [1], Yoshinori Takahashi [2, 4], Neelam Desai [2], Jun Zhang [3], Jacob M. Serfass[1], Yu-Guang Shi [3], Christopher J. Lynch [3], and Hong-Gang Wang [1, 2, 4]

[1] Department of Pharmacology, [2] Department of Pediatrics, [3] Department of Molecular Physiology, [4] Penn State Cancer Institute, Penn State University College of Medicine, Hershey, PA 17033

Supplementary Information

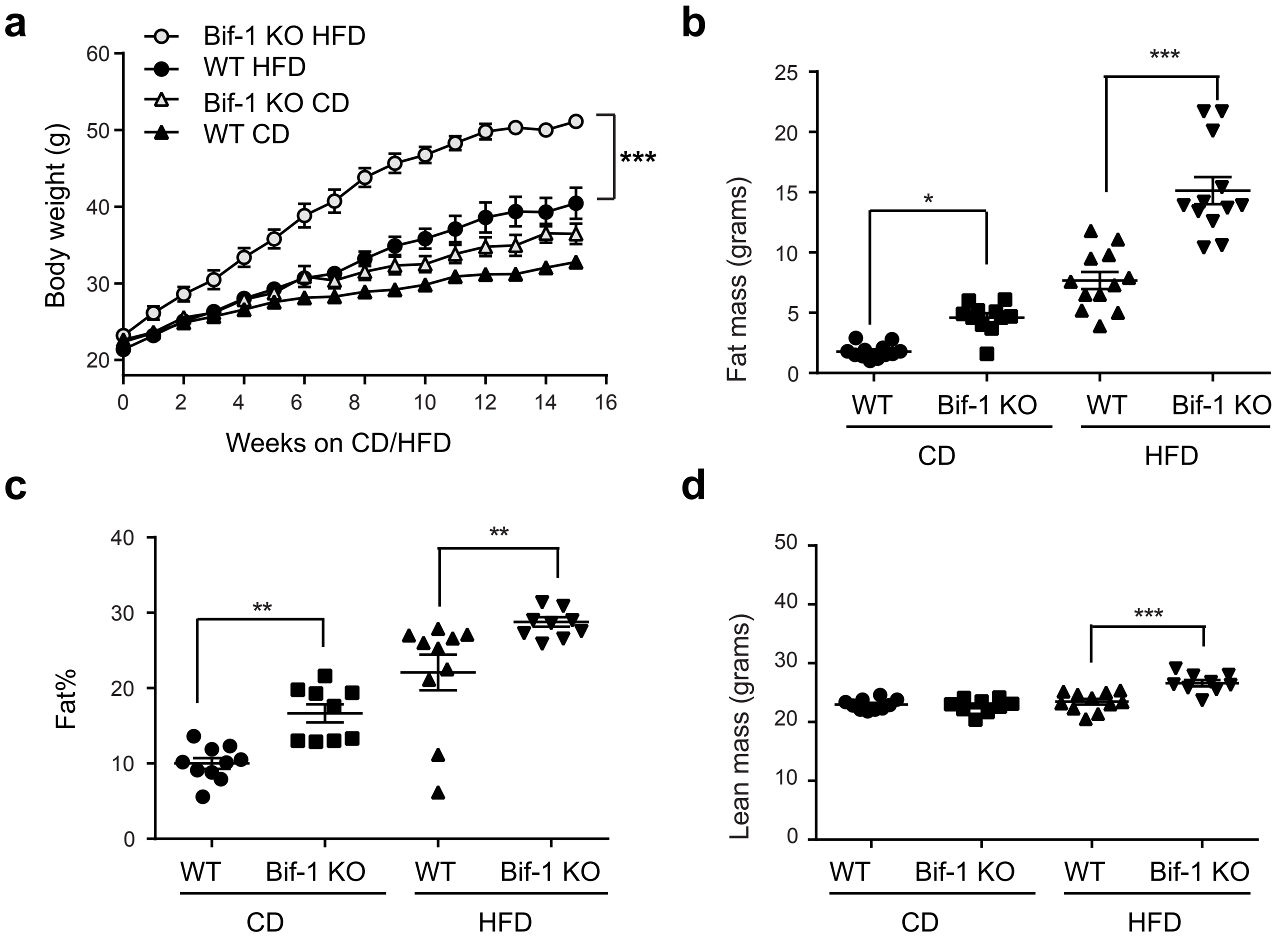

Figure S1. Metabolic characteristics of male mice fed with indicated diet. (**a**) Mice were fed with HFD or control CD starting at 6-week of age and body weight of mice was monitored for 15 weeks. (**b-d**) Loss of *Bif-1* leads to larger total (**b**) and percent (**c**) fat mass and has minimal effect on lean mass (**d**). Body composition of mice fed with the indicated diet for 19 weeks was measured using TD-NMR. Statistical significance was determined using one-way ANOVA followed by Bonferroni's multiple comparison test. Oral glucose tolerance test was performed in 16 h-fasted mice, *n*=9-11. All values are mean ± SEM. Differences with controls were significant for *$p < 0.05$, **$p < 0.01$, and ***$p < 0.001$.

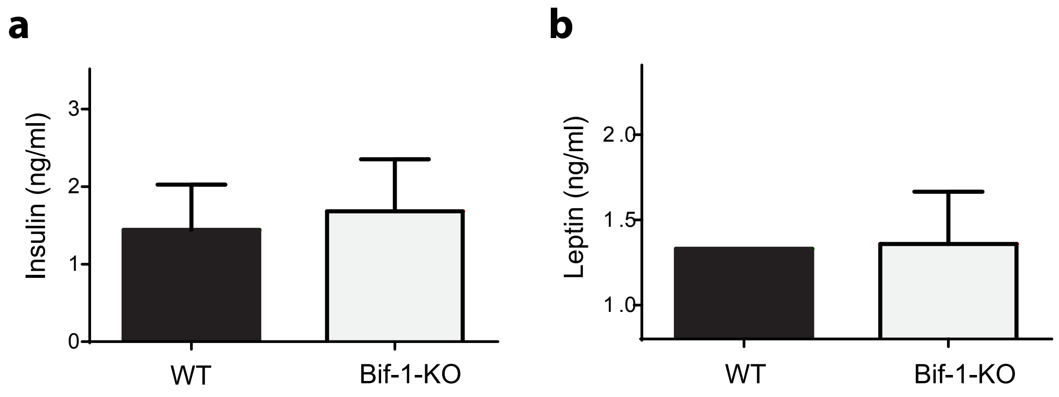

Figure S2. Plasma insulin and leptin levels in 10-week-old mice. Levels of insulin (**a**) and leptin (**b**) in plasma from 10-week-old mice fed with CD were measured by ELISA assays, n=1-2.

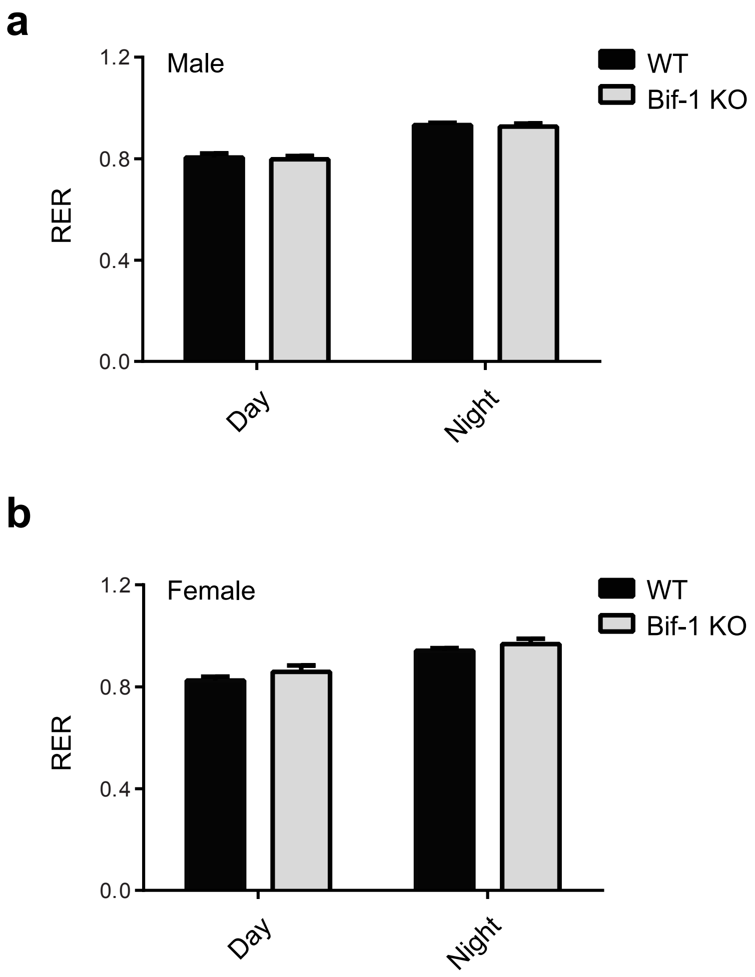

Figure S3. Respiratory exchange ratio (RER) of WT and Bif-1 KO mice. (**a, b**) RER values for male (**a**) and female (**b**) mice were calculated using *V*CO2 and *V*O2 measured during a 48 h-cycle (n=4). All values are mean ± SEM.

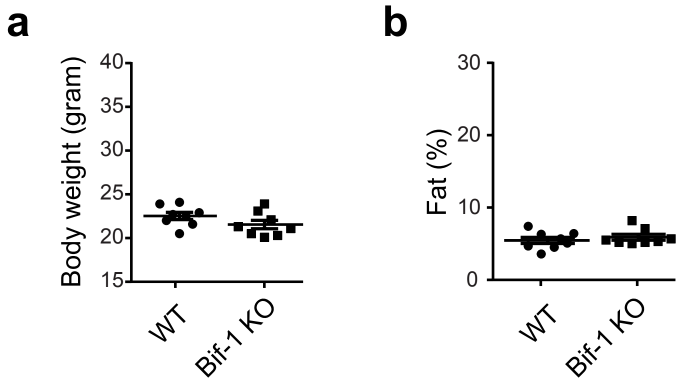

Figure S4. Body weight and fat percentage in 6-week-old mice. (**a**, **b**) Body weight (**a**) and fat percentage over body weight (**b**) in control diet-fed, 6-week-old mice.

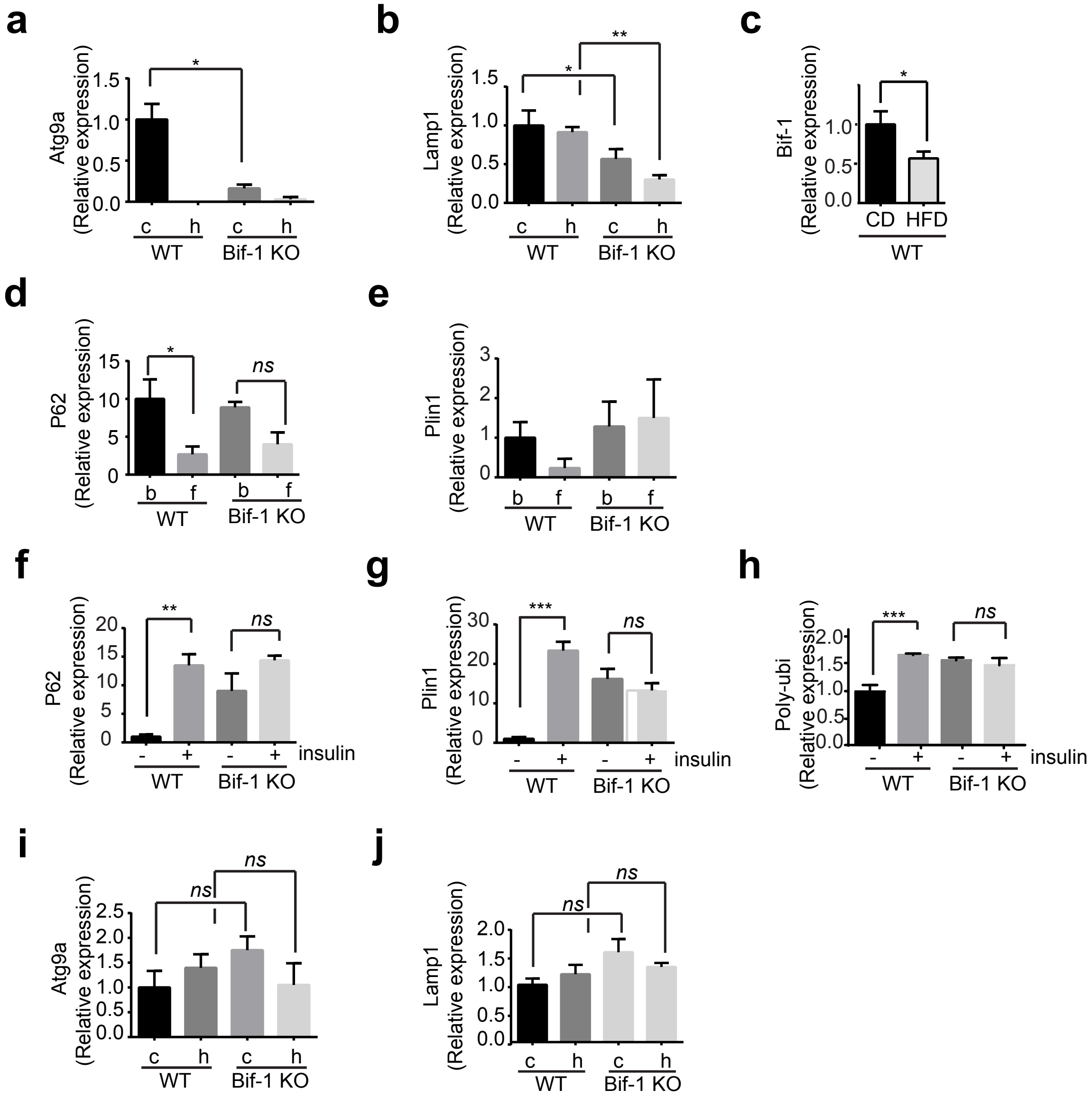

Figure S5. Quantifications for immunoblots in Figure 6. (**a-c**) Quantification for immunoblots in **Figure 6a**. (**d-e**) Quantification for immunoblots in **Figure 6b**. (**f-h**) Quantification for immunoblots in **Figure 6c**. (**j, k**) Quantification for immunoblots in **Figure 6d**. Image J software was used in quantifications and expression of indicated proteins was normalized to β-actin. Statistical significance was determined using Student's *t*-test for single comparison and One-way ANOVA for multiple comparison followed by Bonferroni's or Dunn's multiple comparison test. n=3-4. All values are mean ± SEM. Differences with controls were significant for *p <0.05, **p <0.01, and ***p <0.001.
